# Nanostructured carbon electrode modified with N-doped graphene quantum dots–chitosan nanocomposite: a sensitive electrochemical dopamine sensor

**DOI:** 10.1098/rsos.171199

**Published:** 2017-11-08

**Authors:** Sami Ben Aoun

**Affiliations:** Department of Chemistry, Faculty of Science, Taibah University, PO Box 30002, Al-Madinah Al-Munawarah, Kingdom of Saudi Arabia

**Keywords:** electrochemical sensor, graphene quantum dots, chitosan, screen printed carbon electrode, dopamine

## Abstract

A highly selective and sensitive dopamine electrochemical sensor based on nitrogen-doped graphene quantum dots–chitosan nanocomposite-modified nanostructured screen printed carbon electrode is presented, for the first time. Graphene quantum dots were prepared via microwave-assisted hydrothermal reaction of glucose, and nitrogen doping was realized by introducing ammonia in the reaction mixture. Chitosan incorporation played a significant role towards the selectivity of the prepared sensor by hindering the ascorbic acid interference and enlarging the peak potential separation between dopamine and uric acid. The proposed sensor's performance was shown to be superior to several recently reported investigations. The as-prepared CS/N,GQDs@SPCE exhibited a high sensitivity (i.e. ca. 418 µA mM cm^−2^), a wide linear range i.e. (1–100 µM) and (100–200 µM) with excellent correlations (i.e. *R*^2^ = 0.999 and *R*^2^ = 1.000, respectively) and very low limit of detection (LOD = 0.145 µM) and limit of quantification (LOQ = 0.482 µM) based on *S*/*N* = 3 and 10, respectively. The applicability of the prepared sensor for real sample analysis was tested by the determination of dopamine in human urine in pH 7.0 PBS showing an approximately 100% recovery with RSD < 2% inferring both the practicability and reliability of CS/N,GQDs@SPCE. The proposed sensor is endowed with high reproducibility (i.e. RSD = ca. 3.61%), excellent repeatability (i.e. ca. 0.91% current change) and a long-term stability (i.e. ca. 94.5% retained activity).

## 1. Introduction

Dopamine is among the very important neurotransmitters ensuring inter-neuronal communication in the human central nervous system [1]. It is vital to many neuronal functions like memory, learning, cognition, behaviour, attention, emotion and movement [2]. Abnormal response of dopamine may cause several diseases like epilepsy, schizophrenia, Parkinson's disease and senile dementia [2–4]. This explains the huge efforts deployed during the last three decades for the determination of dopamine using a great deal of techniques including fluorescence spectrometry [5], high performance liquid chromatography [6], capillary electrophoresis [7], UV-visible spectrophotometry [8], liquid chromatography-electrospray tandem mass spectrometry [9] and enzymatic methods [10]. Due to its fascinating electroactivity [11], dopamine determination has been extensively studied by various electrochemical methods [12]. Although the latter are sensitive, rapid, simple and cost-effective, their major drawback lies in the interference of uric acid and ascorbic acid that always coexist with dopamine in biological fluids, with the oxidation potentials of all three being actually very similar [2,13]. Moreover, these interfering species might cause poor reproducibility and selectivity if their oxidation products accumulate on the electrode surface resulting in its fouling [14]. Several attempts have been made to overcome this problem through utilization of several materials like gold nanoparticles [15], gold nanoparticles–polyaniline [16], carbon nanotubes [17], graphene [18] and others. Despite the successful sensing of dopamine using these materials overcoming the selectivity issue, other concerns pertain to sensitivity, lack of easy synthesis protocol and elevated cost. The real challenge is therefore to develop a sensor that is not only reliable, selective and sensitive, but also economical and practical.

Graphene quantum dots (GQDs) emerged recently as a new class of carbon nanomaterial having ‘hybrid’ characteristics of carbon and graphene [19]. They are zero-dimensional closely packed honeycomb graphene nanosheets below 100 nm in size [3]. Additionally, GQDs are endowed with high water solubility, good biocompatibility, low toxicity and excellent electrical conductivity [20,21]. Based on these outstanding properties, GQDs have been applied in various fields such as batteries and capacitors [22], photovoltaics [23], bioimaging [24], drug delivery [25], photocatalysis [26], and chemical, electrochemical and biosensors [27–30]. Nevertheless, GQDs sensitivity and selectivity issues remain a great challenge in the field of sensor applications [31].

Recent works reported that doping with heteroatoms was shown to be a very effective approach in the quest to improve GQDs intrinsic properties, for instance the chemical stability, electrical conductivity and electrocatalytic activity [32,33]. The nitrogen atom, with its comparable number of valence electrons and size with carbon, has been extensively used to prepare nitrogen-doped graphene quantum dots [34,35]. Excellent electrocatalytic activities were obtained, such as in the case of oxygen reduction reaction [36] and H_2_O_2_ reduction [37].

Two main approaches were developed for the preparation on GQDs: top-down and bottom-up. The former involves a nanosize carving of carbon materials via chemical or physical routes [38,39], while the latter is based on the carbonization of an organic precursor by means of a thermal treatment [40]. By far, the bottom-up approach is more advantageous as it allows precise control of size and morphology [41] in addition to the ease of operation, low cost, higher aqueous solubility and purity [42]. Recently, microwave-assisted pyrolysis has been introduced as a rapid, facile, energy-efficient and economic method for the nitrogen-doped graphene quantum dots preparation [43–45].

Screen printed electrodes emerged in the last few years as a new technology for the preparation of electrochemical detection electrodes with high reproducibility. Carbon screen printed electrodes, as cheap and easy to fabricate materials, constitute the major part of prepared and investigated electrodes, so far. Some very interesting results were obtained upon their surface modification, like noble metals, inorganic nanocomposites and enzymes [46–48].

In the present work, a nanocomposite made of chitosan and microwave-assisted nitrogen-doped graphene quantum dots will be used as a surface modifier of a screen printed nanostructured carbon electrode. The synthesized nanocomposite is characterized by morphological and spectrophotometry techniques. The prepared electrochemical platform will be investigated as a potential dopamine sensor using cyclic and differential pulse voltammetries and the evaluated electrochemical sensor parameters will be assessed against previously reported works in the literature. Finally, real sample analysis will be carried out in order to judge the applicability of the presented sensor, and its repeatability, reproducibility and stability characteristics will be investigated.

## 2. Material and methods

### 2.1. Chemicals and materials

Chitosan, dopamine (≥98%), ascorbic acid (≥99%) and uric acid (≥99%) were purchased from Sigma-Aldrich (www.sigmaaldrich.com). Potassium ferrocyanide (≥98%) and potassium ferricyanide (≥99%) were obtained from BDH Chemicals Ltd. (www.bdhme.com). Potassium chloride (≥99%), hydrochloric acid (37%) and ammonia (30%) were purchased from Panreac (www.panreac.com). Sulphuric acid (≥36%) was purchased from Tedia Inc. (www.tedia.com). Acetic acid glacial (≥99.5%) was obtained from ADWIC (www.nasrpharma.com). d-Glucose anhydrous was purchased from Techno Pharmchem (www.technopharmchem.com).

All solutions were prepared with ultrapure water (18.2 MΩ cm) from a Milli-Q water purification system, Millipore (www.merckmillipore.com).

A screen printed three-electrode system (SPCE) from Orion High Technologies (www.orion-hitech.com) comprising a nanostructured carbon working electrode (*ϕ* = 4 mm), a Ag|AgCl reference electrode and a carbon counter electrode was employed in all electrochemical measurements.

### 2.2. Quantum dots preparation

In a typical synthesis protocol, 2 g glucose was dissolved in 20 ml of a four times diluted ammonia solution then transferred into a Discover SP microwave synthesizer, CEM Corp. (www.cem.com) and irradiated under 300 W for 5 min. The solution colour changes from colourless to light brown indicating the formation of nitrogen-doped graphene quantum dots which will be denoted as N,GQDs in this work. The same experiment was repeated in the absence of ammonia for comparison and the prepared graphene quantum dots are denoted as GQDs in this case.

### 2.3. Graphene quantum dots–chitosan nanocomposite preparation

An adequate amount of chitosan (CS) was dissolved in 1% acetic acid solution in order to prepare a 0.5% CS solution in 1% acetic acid, which was subsequently stirred for 2 h until a clear solution was obtained. Then 1 ml of this CS solution was mixed with 1 ml of the as-prepared N,GQDs solution and stirred for a further hour, resulting in the formation of CS/N,GQDs nanocomposite solution.

### 2.4. Electrochemical sensor preparation

The SPCE was rinsed with Milli-Q water, and then electrochemically cleaned by repetitive potential cycling in a 0.5 M H_2_SO_4_ solution at 100 mV s^−1^ scan rate between −1 V and 1 V. The SCPE was then dried under high purity nitrogen atmosphere and 10 µl of the CS/N,GQDs nanocomposite was drop-casted onto the working electrode surface and dried for 1 h at 40°C. The obtained CS/N,GQDs@SPCE was thoroughly washed with a copious amount of Milli-Q water then dried under high purity nitrogen atmosphere.

### 2.5. Electrochemical measurements

A computer-controlled Autolab PGSTAT 128N potentiostat/galvanostat (www.metrohm-autolab.com) was used in all electrochemical experiments that were conducted in a miniaturized 4 ml electrochemical cell. All potentials are reported with respect to the Ag|AgCl reference electrode.

Cyclic voltammetry (CV) measurements were conducted in unstirred, air-saturated conditions and further specific experimental details will be given when appropriate. Electrochemical impedance spectroscopy (EIS) experiments were realized using the integrated FR32 frequency response analyser employing a single sine wave with 10^−2^ V amplitude and a maximum integration time of 0.125 s while maintaining the potential at 200 mV and scanning the frequency in the interval 5 × 10^−2^ Hz–10^5^ Hz. As for differential pulse voltammetry (DPV) measurements, the potential was scanned at a rate of 50 mV s^−1^ with 50 mV modulation amplitude, 5 mV step potential and 50 ms modulation time.

### 2.6. Characterization techniques

Morphological characterization was conducted using a JEM-1400 transmission electron microscope (TEM), JEOL Co. (www.jeol.co.jp), operating at acceleration voltage of 98** **kV. The spectroscopic characterization was done by recording the absorbance spectra with an Evolution 201 UV-visible spectrophotometer, Thermofisher Scientific Inc. (www.thermofisher.com), and the photoluminescence (PL) spectra were measured by means of an RF-5301PC spectrofluorophotometer from Shimadzu Scientific Instruments (www.ssi.shimadzu.com).

## 3. Results and discussion

### 3.1. N,GQDs characterization

**Figure 1.**
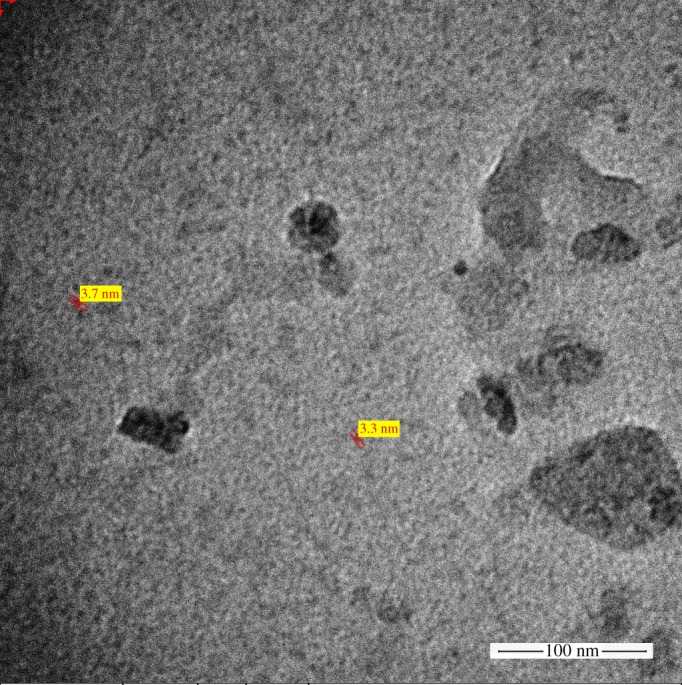
TEM image of the prepared N,GQDs.

The prepared quantum dots showed a monodispersed, spherical shape with a size ranging between approximately 3 and 4 nm as revealed from the TEM image shown in figure 1 which is in the typical range of nitrogen-doped graphene quantum dots [49].

**Figure 2.**
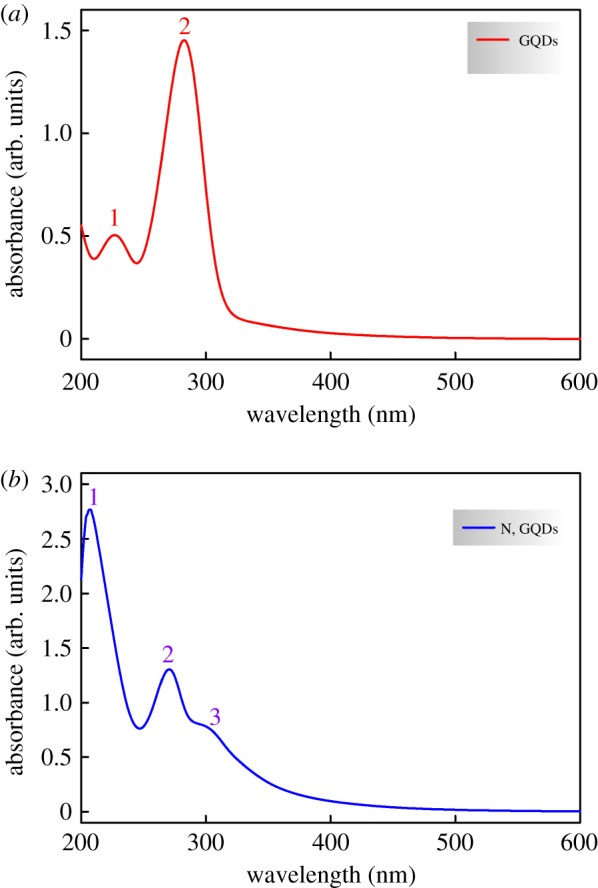
UV-Vis absorbance spectra of the prepared (*a*) GQDs and (*b*) N,GQDs.

On the other hand, the formation of graphene quantum dots is further confirmed by UV-Vis spectra (cf. figure 2*a*) showing two absorbance peaks at 227 nm and 383 nm, typical of an aromatic system, associated to *π* → π* and *n *→ π* electron transitions of C═C and C═O, respectively [50,51]. Moreover, the successful nitrogen incorporation into the graphene quantum dots matrix is also confirmed by inspection of the spectra shown in figure 2*b*, exhibiting three adsorption peaks. The first two situated at 207 nm and 271 nm are respectively ascribed to *π*/*n *→ π* electron transitions of C═C and C═O while the third one occurring at 302 nm corresponds to the C═N *π *→ π* electron transition [52,53].

**Figure 3.**
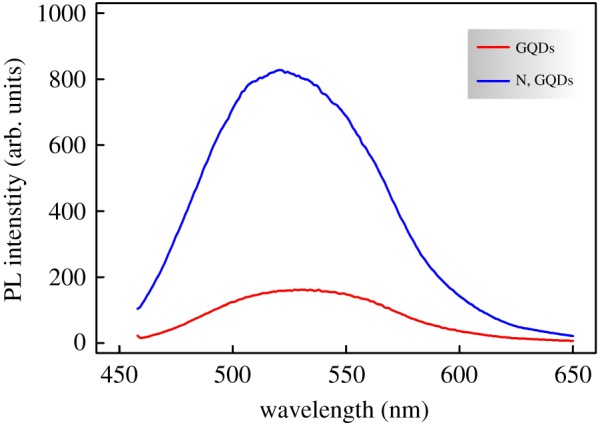
PL emission spectra of the prepared GQDs and N,GQDs.

It is very well reported in the literature that an enhanced photoluminescence of nitrogen-doped graphene quantum dots is exhibited compared with their nitrogen-free counterparts [54,55], therefore the prepared GQDs and N,GQDs PL spectra were recorded and results are shown in figure 3. The presented data are for the maximum emission that was obtained with an excitation wavelength *λ*_ex_ = 450 nm. The drastically increased photoluminescence (blue line) confirms again the successful nitrogen doping of the prepared quantum dots. It is noteworthy that figure 3 shows a PL emission's 10 nm blue shift for the N,GQDs compared to GQDs (i.e. emission peak shift from 531 to 521 nm) attributable to the increased electron affinity upon incorporation of nitrogen atoms [56].

### 3.2. Electrochemical behaviour of the prepared CS/N,GQDs@SPCE

**Figure 4.**
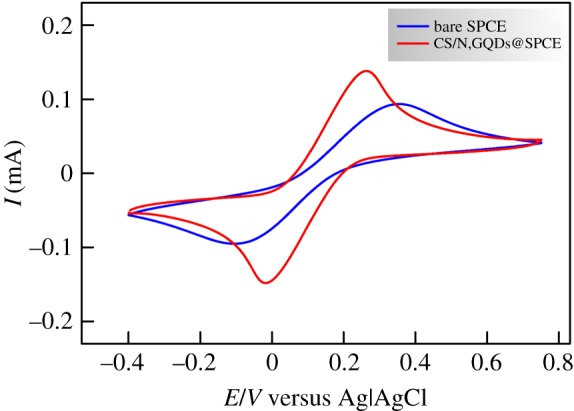
CVs of bare SPCE and CS/N,GQDs@SPCE in 0.1 M KCl solution containing 5 mM [Fe(CN)_6_]^3−/4−^ with 50 mV s^−1^ scan rate.

In the present work, the redox couple [Fe(CN)_6_]^3−/4−^ was used as an electrochemical probe for monitoring the interfacial properties variations upon modification of bare SPCE and the preparation of the CS/N,GQDs@SPCE electrochemical sensor. A general overview is obtained from CV experiments (cf. figure 4) showing a clear enhancement of the electrochemical properties of the modified electrode. For instance, as compared to bare SPCE, the CS/N,GQDs@SPCE voltammogram shows a significant improvement of the anodic and cathodic peak currents with a very-close-to-unity ratio in addition to smaller peak separation owing to an improved electron transfer rate, a larger electroactive surface area and probably a better conductivity of the electrode surface.

**Figure 5.**
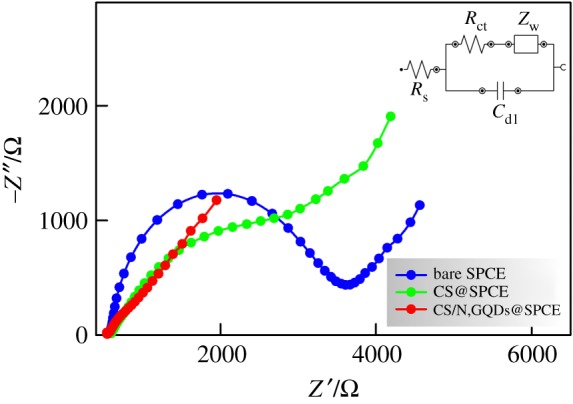
Nyquist plots of bare SPCE, CS@SPCE and CS/N,GQDs@SPCE in 0.1 M KCl solution containing 5 mM [Fe(CN)_6_]^3−/4−^.

A more effective electrochemical characterization was conducted by EIS and typical Nyquist plots are given in figure 5 showing two portions at high and low frequencies. The former (i.e. a semicircular shape) reveals the electron transfer limited process, while the latter (i.e. a linear branch) is related to the diffusion controlled process [57]. The experimental data were best fitted to the equivalent circuit given in the inset of figure 5 [58].

*R*_s_ represents the solution resistance, *R*_ct_ represents the charge transfer resistance which is inversely proportional to the electron transfer rate and corresponds to the diameter of the semicircular loop of the plot, *C*_dl_ represents the double layer capacitance and *W* is a Warburg impedance used to fit the linear portion of the Nyquist plot and is an indication of the diffusion process. An eye-catching feature is the impressive decrease of the semicircle loop's diameter in the case of CS/N,GQDs@SPCE, which not only confirms the surface modification and successful attachment of the CS/N,GQDs nanocomposite but also reveals a significant increase of the charge transfer rate as a consequence of the great decrease of *R*_ct_. In this respect, the fitting results show a great decrease in charge transfer resistance from ca. 2631 Ω to ca. 159 Ω which goes in line with a tremendously increasing value of *C*_dl_ from ca. 1.9 µF to 44 µF, confirming the excellent improvement of electrical conductivity. It is worth mentioning here that in the absence of N,GQDs, the modification of the SPCE with CS resulted in a significant improvement of the electrode conductivity lying in between that of the bare SPCE and CS/N,GQDs@SPCE (cf. CS @SPCE plot in figure 5). In this respect, the obtained values of *R*_ct_ and *C*_dl_ were 1637 Ω and 22 µF, respectively. Such results prove that the observed enhancement of the electrochemical properties of the prepared CS/N,GQDs@SPCE are indeed a combination of CS and N,GQDs induced effects.

### 3.3. Cyclic voltammetry behaviour of dopamine at the prepared CS/N,GQDs@SPCE

**Figure 6.**
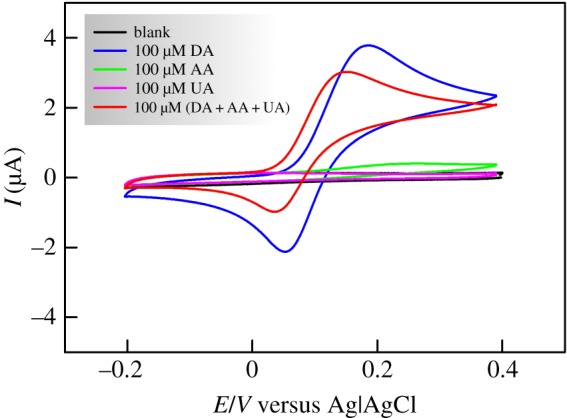
CVs of bare SPCE in 0.1 M PBS solution in the absence and presence of dopamine (DA), ascorbic acid (AA), uric acid (UA) and a mixture of (DA + AA + UA), 100 µM each, with 100 mV s^−1^ scan rate.

**Scheme 1.**
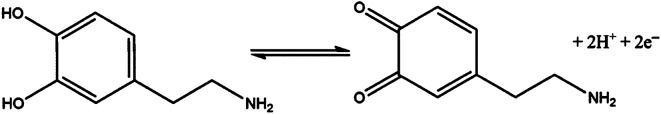
The redox reaction of dopamine to/from dopamine-o-quinone.

In the following, all electrochemical measurements were conducted in 0.1 M pH 7.0 PBS solution. Figure 6 shows the CVs of bare unmodified SPCE in the absence and presence of 100 µM dopamine (DA). At first sight, this nanostructured electrode seems to perform quite well with the appearance of well-defined peaks for the oxidation and reduction of DA as per the reaction equation outlined in scheme 1. Dopamine is oxidized during the positive going scan to dopamine-o-quinone and the latter is reduced back to dopamine in the course of the negative going scan.

This nice-looking CV is completely altered upon addition of 100 µM uric acid (UA) and/or ascorbic acid (AA). Knowing that these are the most common species interfering with DA [59], this represents a serious drawback when it comes to DA sensing in body fluids. Although UA itself shows almost no redox behaviour in the present work conditions and AA shows insignificant oxidation current at the bare SPCE (cf. figure 6), their presence with DA results in a huge decrease of both oxidation and reduction currents which would lead to erroneous DA detection.

**Figure 7.**
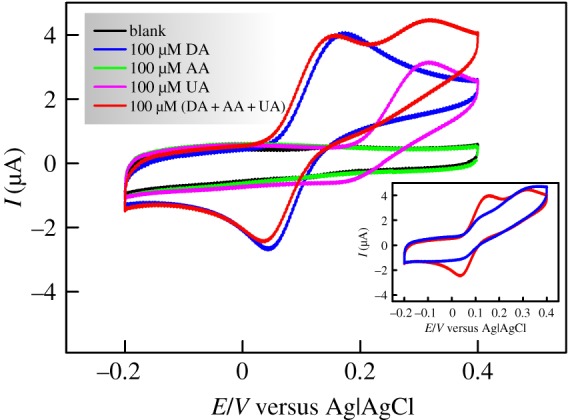
CVs with 100 mV s^−1^ scan rate of CS/N,GQDs@SPCE in 0.1 M PBS solution in the absence and presence of DA, AA, UA and a mixture of (DA + AA + UA), 100 µM each, with an inset showing the comparison of CS@SPE (blue) and CS/N,GQDs@SPCE (red).

On the other hand, when SPCE was modified with the prepared CS/N,GQDs nanocomposite, a totally different behaviour was observed as displayed in figure 7. In detail, AA redox reaction is completely hindered against a clear improvement of DA both anodic and cathodic currents in addition to a nice peak separation of DA and UA. The observed change in electrochemical activities can be explained by the following two synergistic phenomena brought about by the modification of the SPCE surface with the as-prepared CS/N,GQDs nanocomposite. (i) The electrostatic repulsion/attraction induced by chitosan. For instance, in pH 7.0 PBS, CS (p*K*_a_ = 6.3), AA (p*K*_a_ = 4.1) and UA (p*K*_a_ = 5.8) are negatively charged while DA (p*K*_a_ = 8.9) is positively charged [60,61]. This would lead to a mutual electrostatic attraction between DA and CS in contrast to an electrostatic repulsion between CS-UA and CS-AA [62], the latter being perhaps stronger, most probably due to larger p*K*_a_ values difference (i.e. 2.2 versus 0.5). (ii) A significantly different π–π interaction between the sp^2^ conjugated carbon–carbon bonds in GQDs and their counterparts in DA, UA and AA. Arranging these π–π interactions in an increasing order of strength would be GQDs-AA < GQDs-UA < GQDs-DA according to the respective structures of these compounds. For instance, the strongest π–π interaction in the case of GQDs-DA is due to the phenyl moiety of DA while the weakest is expected for GQDs-AA since AA structure comprises less π bonds compared to UA [62].

This synergy is supported by comparison of the CVs of CS@SPE and CS/N,GQDs@SPCE shown in the inset of figure 7. We can clearly notice the significant enhancement of DA redox peaks at CS/N,GQDs@SPCE that are otherwise hardly noticeable in the case of CS@SPE due to interfering UA and AA. This comes in addition to a distinguishable oxidation peak separation of DA and UA.

### 3.4. Dopamine electrochemical sensing performance at the prepared CS/N,GQDs@SPCE

Further investigations were carried out in the quest for a possible electrochemical sensing application of the as-prepared CS/N,GQDs@SPCE. For this, DPV was used as a fast, effective and a more sensitive electrochemical technique compared to CV [63].

**Figure 8.**
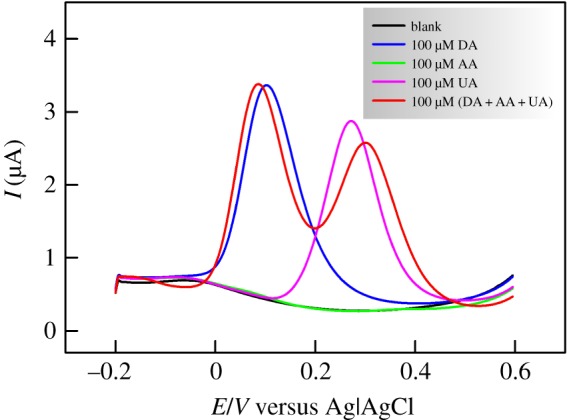
DPVs of CS/N,GQDs@SPCE in 0.1 M PBS solution in the absence and presence of 100 µM DA, 100 µM AA, 100 µM UA and a mixture of (DA + AA + UA), 100 µM each.

Figure 8 shows the DPV results of the as-prepared CS/N,GQDs@SPCE for the same electrolytic composition as in the previous section with a narrow focusing on the oxidative voltammograms only as per the discussed CV data. One can clearly notice the complete hindrance of AA oxidation current (i.e. nearly identical to the blank) and the very well defined peaks for both DA and UA oxidation that are clearly separated (i.e. Δ*E*_p_ = ca. 171 mV). Interestingly, when all three analytes are present together, DA peak current retained its original value (i.e. ca. 0.4% change) while the peak potential shifted to less anodic values against an opposite shift of UA peak resulting in ca. 46 mV enlargement of the peak-to-peak separation (i.e. reaching ca. 217 mV). On the other hand, the peak current for UA oxidation decreased noticeably (i.e. ca. 12%) in the presence of interfering DA and AA. The obtained results reveal the high selectivity of the prepared system to DA, which is of prime importance for electrochemical sensing.

**Figure 9.**
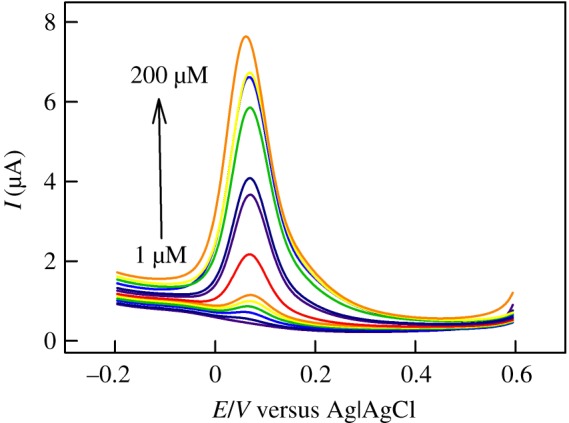
DPVs of CS/N,GQDs@SPCE in 0.1 M PBS solution in the presence of increasing concentrations of DA.

The second wing of the sensor is the sensitivity, which is evaluated through determining the relationship between the DA concentration and the DPV peak current in an extended concentration range. The obtained data are shown in figure 9 in the concentration range 1–200 µM, exhibiting a steady peak current increase with increasing DA concentrations.

**Figure 10.**
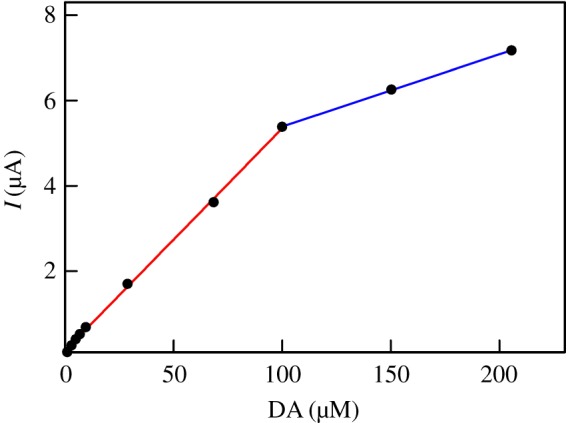
Variations of DPV peak currents with DA concentrations as extracted from figure 9.

Plotting the extracted values of peak current as a function of DA concentration gives straight lines in the ranges 1–100 µM and 100–200 µM with excellent correlations (i.e. *R*^2^ = 0.999 and *R*^2^ = 1.000, respectively) as shown in figure 10. From the slope of the first linear branch, the sensitivity (*S*) was evaluated to be *S* = ca. 418 µA mM^−1^ cm^−2^. On the basis of signal-to-noise ratios of 3 and 10, the limit of detection (LOD) and the limit of quantification (LOQ) were evaluated, respectively. The estimated values are LOD = 0.145 µM and LOQ = 0.482 µM.

The current electrochemical sensor performance was compared to other very recently reported results, as summarized in table 1, showing the great competitiveness of the proposed sensor.

**Table 1.** Comparison of the prepared DA electrochemical sensor's performance to recent literature.

electrode	linear range (μM)	detection limit (μM)	reference
GN/PANI/Au/GCE	0.07–1.05 and 1.47–5.24	0.024	[64]
AgNP/SiO_2_/GO/GCE	2–80	0.26	[65]
F-CuInS_2_ QDs	0.5–40	0.2	[66]
TC8A/Au	1–100	0.5	[67]
AuNS/GCE	2–298	0.28	[68]
Laccase/SiO_2_-PA/GCE	1–103	0.26	[69]
CS/N,GQDs@SPCE	1–100 and 100–200	0.145	the present work

### 3.5. Application to real sample analysis

The practicability of the prepared electrochemical sensor for the detection of DA in real samples was tested using urine samples from a healthy individuals. The collected samples were hundred-times diluted in PBS solution to overcome the urine matrix effect interferences [12]. Initially, no DA was detected, then consecutive DA additions were performed and recovery percentages were calculated based on the determined DA concentrations. The results are summarized in table 2. The displayed results show very good recovery (i.e. approx. 100%) and very reasonable relative standard deviations (i.e. less than 2%), indicating a promising applicability with a good reliability of the prepared sensor for the determination of DA in real samples.

**Table 2.** DA determination in urine samples at CS/N,GQDs@SPCE.

sample	initial [DA] (*I*)	added [DA] (*A*)	found [DA] (*F*)	recovery % (*R* = 100×[*I* + *A*/*F*])	RSD% (3 runs)
1	not detected	30 µM	29.7 µM	99.14	0.64
2	not detected	50 µM	50.2 µM	100.47	1.60

### 3.6. Reproducibility, repeatability and stability of the prepared CS/N,GQDs@SPCE sensor

Using six different CS/N,GQDs@SPCE sensors revealed highly reproducible results with very reasonable relative standard deviation (i.e. RSD = ca. 3.61%).

The prepared electrochemical sensor showed an excellent repeatability with only ca. 0.91% current change after twelve consecutive electrochemical runs, proving the reliability of the current signal.

We also investigated the stability and long-term reusability of the proposed dopamine sensor by monitoring its electrochemical activity over a one-month period. The results were very impressive as we found that the sensor retained ca. 94.5% of its peak current intensity.

## 4. Conclusion

In the present work, a highly selective dopamine electrochemical sensor was developed based on the modification of a nanostructured carbon screen printed electrode with a chitosan/nitrogen-doped graphene quantum dots nanocomposite. The successful preparation of N,GQDs was confirmed by TEM surface analysis and UV-Vis spectrophotometry and supported by the shown high photoluminescence compared to N-free GQDs. Chitosan addition showed a significant impact in avoiding the commonly reported interferences with ascorbic acid and uric acid. EIS confirmed the superior electrochemical properties of the prepared CS/N,GQDs@SPCE in comparison to bare SPCE. For instance there was a marked decrease of charge transfer resistance (i.e. from ca. 2631 Ω to ca. 159 Ω) and by consequence a promoted electron transfer kinetics. The high sensitivity (*S* = ca. 418 µA mM^−1^ cm^−2^) of the proposed sensor in addition to its low detection limit (LOD = 0.145 µM) and large dynamic range (1–200 µM) puts it in the forefront of the very recently developed dopamine sensors. In addition, real samples analysis showed very promising results based on DA detection in urine samples with high recovery and low relative standard deviation percentages (i.e. approximately 100% and less than 2%, respectively).

## Data accessibility

All data relevant to this work are presented throughout the paper.
